# Mortality gap and physical comorbidity of people with severe mental disorders: the public health scandal

**DOI:** 10.1186/s12991-021-00374-y

**Published:** 2021-12-13

**Authors:** Andrea Fiorillo, Norman Sartorius

**Affiliations:** 1 Department of Psychiatry, University of Campania “L Vanvitelli”, Naples, Italy; 2 Association for the Improvement of Mental Health Programmes, Geneva, Switzerland

**Keywords:** Mortality, Physical comorbidity, Life expectancy, Severe mental disorder, Public health

## Abstract

**Background:**

Patients suffering from severe mental disorders, including schizophrenia, major depression and bipolar disorders, have a reduced life expectancy compared to the general population of up to 10–25 years. This mortality gap requires urgent actions from a public health perspective in order to be reduced.

**Main text:**

Factors associated with the high mortality rates in patients with severe mental disorders can be grouped into four groups: those related to the patients, to psychiatrists, to other non-psychiatrist medical doctors and to the healthcare system. Each of these factors should become the target of specific and dedicated interventions, in order to reduce the morbidity and mortality rate in patients with severe mental disorders. All these elements contribute to the neglect of physical comorbidity in patients with severe mental. In particular, the long-standing separation of psychiatry from other branches of medicine and the lack of specific training on this issue further contribute to the poor attention dedicated to management of physical comorbidities. Recently, several professional associations have invited national bodies regulating education of healthcare professionals to include the management of physical health of people with severe mental disorders in undergraduate and postgraduate educational programs.

**Conclusions:**

The premature mortality in patients with severe mental disorders is a complex phenomenon resulting by the interaction of several protective and risk factors. Therefore, a multilevel approach is needed, in which the different stakeholders involved in health care provision establish workforces for the long-term management of physical and mental health conditions.

## Background

Patients suffering from severe mental disorders, such as schizophrenia, major depression and bipolar disorders, have a reduced life expectancy compared to the general population of up to 10–25 years [1, 2]. This “mortality gap” between the general population and patients with severe mental disorders has been rightly described as a “public health scandal” [3, 4], requiring urgent actions from healthcare professionals and governments worldwide. Life expectancy is substantially grown in high-income countries, due to medical advancements, improvements in hygiene and food supply, but it remains unacceptably low in lesser income countries [5–8].

## Main text

This high mortality rate is not due to mental illness per se, rather it is the consequence of the simultaneous presence of comorbid physical health problems, such as cardiovascular, respiratory, metabolic, infectious diseases and cancer [9–12]. In addition, people with severe mental disorders are at higher risk of developing obesity and metabolic syndrome than the general population—with a prevalence rate of these conditions ranging from 40 to 70% in patients with schizophrenia and 20–30% in persons with bipolar disorders [13–18].

Factors associated with the high mortality rates in patients with severe mental disorders can be grouped into four groups: those related to the patients, to psychiatrists, to other non-psychiatrist medical doctors and to the healthcare system [19]. Factors related to patients include some of the characteristics of mental disorders such as the decline in cognitive functioning [20, 21], the patient’s reluctance to attend check-up visits [22–26], the neglect of physical health needs by patients and their caregivers [27], and the adoption of unhealthy lifestyle behavior [28–31], including heavy smoking [32–34], unbalanced diet [35], and substance abuse [36–39]. Another factor contributing to the higher mortality rate in this patient group is the intake of some antipsychotic and antidepressant medications which have metabolic side effects [40–51]. Psychiatrists frequently focus on mental rather than on physical health of their patients, rarely undertake physical examinations and often have poor communication and collaboration with primary care physicians or other clinicians, partly due to the long-standing separation of psychiatric departments from other medical wards or hospitals [52–55]. Conversely, non-psychiatric clinicians frequently have a negative attitude towards people with mental disorders, underestimating the seriousness of their complaints of signs of physical illness [56–58]. Healthcare-related factors also hamper care for physical health of patients with severe mental disorders: there is often no clarity about the person or the team who are responsible for detecting and managing physical problems in patients with severe mental disorders and the lack of access to health care services for patients with severe mental disorders is hampered [59, 60]. The excessive division and over-specialization of medical disciplines and the fragmentation of medical knowledge [61–63] also play an important role: some psychiatrists only deal with specific age groups (e.g., adolescents or the elderly) or specific diseases (e.g., only with eating disorders) or stages in disease-development (e.g., early intervention services) [64].

The neglect of physical comorbidity in patients with severe mental disorders is in part due to long-standing separation of psychiatry from other branches of medicine and also to the lack of attention to comorbidity during the undergraduate and postgraduate training of psychiatrists and other medical specialists [65–68]. For a long time, the prevalence of comorbidity has been underestimated by health care professionals and—on the other hand—patients have been reluctant to speak to psychiatrists about their physical health problems. Recently, professional associations such as the Royal College of Psychiatrists, the Royal College of Practitioners, the European Psychiatric Association (EPA) and the World Psychiatric Association (WPA) as well as the World Health Organization (WHO) invited national bodies regulating education of healthcare professionals to include the management of physical health of people with severe mental disorders in undergraduate and postgraduate educational programs [69–71]. A revision of educational requirements for healthcare professionals to improve their training curricula has also been proposed in order to adequately prepare the young generation of professionals to manage physical comorbidities in patients with severe mental disorders. In particular, the WPA, the world’s largest professional association of psychiatrists and mental health professionals [72, 73], has recently established a WPA Working Group on Physical Comorbidities [74, 75], so to promote attention to management of physical comorbidities in people with severe mental disorders, to update training materials available on the educational portal of the Association [76], to organize webinars and seminars with international experts in the field [77–79] and to promote the dissemination of good clinical practice for improving physical health in patients with severe mental disorders [80–83].

## Conclusion

The WHO has recently developed international guidelines on how to improve physical health in people with severe mental disorders [84]. According to the WHO, the premature mortality is a complex phenomenon resulting by the interaction of several protective and risk factors; therefore, a multilevel approach is needed, in which the different stakeholders involved in health care provision establish workforces for the long-term management of physical and mental health conditions [85–87]. The different stakeholders would include policy-makers, psychiatrists, other medical specialists, users, and carers of people with mental disorders [88]. The actions to be implemented include control of risk factors, scaling up management in primary health care, and development of national policies. At the level of scaling up management of primary health care, the education of clinicians, general practitioners and early career psychiatrists represent an essential element to really challenge the “scandal of premature mortality” in people with mental disorders [89, 90].

## Data Availability

Not applicable.

## Abbreviations

WHO: World Health Organization
WPA: World Psychiatric Association
EPA: European Psychiatric Association
